# 3D Hand Pose Estimation Based on Five-Layer Ensemble CNN

**DOI:** 10.3390/s21020649

**Published:** 2021-01-19

**Authors:** Lili Fan, Hong Rao, Wenji Yang

**Affiliations:** 1 School of Information Engineering, Nanchang University, Nanchang 330031, China; 411014519016@email.ncu.edu.cn; 2 Center of Computer, Nanchang University, Nanchang 330031, China; raohong@ncu.edu.cn; 3 School of Software, Jiangxi Agricultural University, Nanchang 330045, China; 4 State Key Lab of CAD & CG of Zhejiang University, Hangzhou 310058, China

**Keywords:** hierarchical thinking, 3D hand pose estimation, RGB image, hand topology

## Abstract

Estimating accurate 3D hand pose from a single RGB image is a highly challenging problem in pose estimation due to self-geometric ambiguities, self-occlusions, and the absence of depth information. To this end, a novel Five-Layer Ensemble CNN (5LENet) is proposed based on hierarchical thinking, which is designed to decompose the hand pose estimation task into five single-finger pose estimation sub-tasks. Then, the sub-task estimation results are fused to estimate full 3D hand pose. The hierarchical method is of great benefit to extract deeper and better finger feature information, which can effectively improve the estimation accuracy of 3D hand pose. In addition, we also build a hand model with the center of the palm (represented as Palm) connected to the middle finger according to the topological structure of hand, which can further boost the performance of 3D hand pose estimation. Additionally, extensive quantitative and qualitative results on two public datasets demonstrate the effectiveness of 5LENet, yielding new state-of-the-art 3D estimation accuracy, which is superior to most advanced estimation methods.

## 1. Introduction

The gesture is among the most commonly used expressions by humans, and accurate 3D hand pose estimation has already become a key technology in the fields of Human-Computer Interaction (HCI) and Virtual Reality (VR) [1,2,3,4,5]. It can help humans communicate with machines in a more natural way. Though 3D hand pose estimation has achieved significant progress after years of research [6,7,8,9,10,11], it is still far from a solved problem due to the challenges of high joint flexibility, local self-similarity, and severe self-occlusion.

At present, mainstream 3D hand pose estimation methods can be classified into two categories: holistic estimation method based on the hand [12,13,14,15,16,17,18,19,20] and hierarchical estimation method based on hand structure [21,22,23,24,25,26]. The holistic estimation method based on the hand aims to directly use a complete hand structure for estimation, which has developed into a mainstream method in recent years [12,14]. For instance, Zimmermann et al. [12] took the whole hand as input and used CNNs to estimate 3D coordinates of 21 keypoints. Spurr et al. [14] trained encoder–decoder pairs from the generative perspective, which allows the estimation of full 3D hand pose from different input modalities. However, these methods fail to make good use of hand structure and lose a high quantity of underlying information concerning hand structure. Thus, some researchers have carried out studies based on hand structure stratification [21,22,26], leveraging hierarchical network to explore the structure of the hand and decomposing the task of 3D hand pose estimation into several sub-tasks, then representative features are extracted through the mutual promotion among tasks, which can effectively improve estimation performance. For example, Guo et al. [21] designed an REN network based on hierarchical thinking, which simply partitioned the extracted feature map into several regions, then estimated the local pose of each region, respectively, and finally merged them into a full hand pose. Zhou et al. [22] divided hand structure into three layers according to the functional importance of different fingers to achieve the estimation of 3D hand pose. Chen et al. [26] proposed a pose guided structured region ensemble network (Pose-REN) to estimate 3D hand pose hierarchically and iteratively. In summary, these methods all exploit the underlying information of hand topology to successfully extract more representative hand features, thereby promoting more accurate hand pose estimation.

Although the above methods [21,22,26] have achieved relatively accurate 3D hand pose estimation, they are all based on depth images. However, depth images are not as universal as RGB images in reality and have low practical applicability. Therefore, it is necessary to develop a hierarchical estimation method based on hand structure for RGB images. Inspired by [22,26], a novel Five-Layer Ensemble CNN (5LENet) for 3D hand pose estimation from a single RGB image is proposed, in which 3D hand pose estimation is decomposed into five 3D single-finger pose estimations, and then the estimates of fingers are fused to estimate a more accurate 3D hand pose. Through effectively utilizing the structural characteristics of the hand to extract deeper and more representative finger feature information, this method can not only promote more accurate 2D finger pose estimation but also can further optimize 3D finger pose estimation, and finally achieve the improvement of 3D hand pose estimation accuracy. The major contributions of this paper can be summarized as follows:

- A 5LENet for 3D hand pose estimation from a single RGB image is proposed, in which hand pose estimation is decomposed into five single-finger pose estimations by using hierarchical thinking. More representative fingers are extracted for estimating a more accurate 3D finger pose, and then the features generated in the process of 3D finger pose estimation are fused to estimate a full 3D hand pose. It can not only extract more effective features but also enhance the association between fingers through the fusion of finger features.
- Five 3D finger pose constraints are newly added, which can not only promote 3D finger pose estimation but also can form soft constraints on 2D finger pose estimation to further indirectly optimize the accuracy of 3D hand pose estimation.
- According to the topology of the hand, a model of the hand with the Palm and middle finger connected is built. This is because the middle finger is located in the middle of five fingers, and its connection with the Palm is tighter. Therefore, we connect the Palm to middle finger, which can successfully solve the accuracy degradation problem caused by connecting the Palm with multiple fingers.
- We conduct experiments on the two public datasets, and results demonstrate that our approach achieves a new state-of-the-art in 3D hand pose prediction, which proves the effectiveness and advancement of 5LENet.

## 2. Related Works

Vision-based 3D hand pose estimation plays an important role in the field of Augmented/Virtual Reality (AR/VR) and Human–Computer Interaction (HCI). Looking back at the work of previous scholars, we can divide the methods for 3D hand estimation into two categories: (1) holistic estimation method based on the hand; (2) hierarchical estimation method based on hand structure.

The holistic estimation method based on the hand is currently mainly based on CNNs, such as Zimmermann et al. [12] who firstly applied CNN for 3D hand pose based on CNN for a single RGB image. The method is composed of three independent networks: the first network performed image segmentation and hand location, the second network performed 2D hand keypoint detection, and the third network performed 2D–3D lifting to derive 3D keypoints. However, their 3D hand pose estimation only relies on 2D keypoints, ignoring rich texture features and latent space information of the RGB image, which would affect the estimation performance to a certain extent. In addition, each network is trained separately, which is prone to falling into local optimization. Wang et al. [13] estimated 2D keypoint features with RGB image features and introduced a channel domain attention mechanism as a weighting scheme to reweight the concatenated features, which can exploit features more efficiently to estimate 3D hand pose. In order to solve the problem of local optimization in literature [12], Liu et al. [27] presented a three-stage cascaded CNN, which realized the mutual promotion between estimation stages, so as to achieve global optimization. Some researchers proposed different solutions to the problem of difficulty in obtaining ground truth 3D keypoints. Cai et al. [15] proposed an estimation method based on weakly-supervised deep learning, in which the estimated 3D hand pose from the RGB image is converted into a depth map through introducing a depth regularizer. Then, the estimated depth map is used to perform weak supervision on the 3D hand pose regression; it can effectively overcome the difficulty of obtaining ground truth 3D annotations, but the method severely relies on the paired RGB and depth images. Based on [15], Chen et al. [19] added a depth-map reconstruction module, which employed a conditional generative adversarial network (GAN) model for generating pseudo-real depth images of color images, then the paired color and pseudo-real depth images were used as input to the hand pose estimation module. Though it eliminates the need for real depth images, it still has a certain domain gap between pseudo-real and real depth images, which leads to low estimation accuracy. Since full 3D mesh information of hand surface is beneficial for the estimation of 3D hand pose, some scholars also integrate mesh information into 3D hand pose estimation. For example, Ge et al. [20] introduced a Graph CNN to estimate 3D hand meshes, in which the extracted hand features and keypoint heatmaps were used as its input, then the estimated mesh information was used to regress 3D hand pose. Though this work achieves a high estimation precision and provides a new direction for the research of 3D hand pose, the ground truth 3D hand mesh is rather rare in the existing datasets.

The abovementioned methods have improved the estimation performance of 3D hand pose to a certain extent, but they all have their problems. Inspired by the multi-task information-sharing mechanism [28], the hierarchical estimation method based on hand structure has become popular in 3D hand pose estimation. It decomposes the task of 3D hand pose estimation into several sub-tasks through employing a hierarchical network to analyze and explore the structure of the hand, then each sub-task learns parallelly based on the shared representation, and finally information from sub-tasks is shared to improve estimation performance. For example, based on the finger functional importance, Zhou et al. [22] designed a three-layer network including the thumb layer, the index finger layer, and the remaining fingers layer, then the local pose of the corresponding layer was estimated separately, and finally the local poses were merged to estimate a full 3D hand pose. Madadi et al. [23] firstly proposed a hierarchical method dividing hand features into six layers based on hand structure, where each layer was associated with a local pose containing a specific single finger, and the remaining layer was used to regress Palm viewpoint. Then, they fused all the features of the last convolutional layers to extract higher-level feature information to estimate 3D global hand pose. Du et al. [24] leveraged the characteristics of related sub-tasks that can facilitate the learning of the main task; they employed a hierarchical model to decompose the hand pose estimation task into two related sub-tasks, palm pose estimation task and finger pose estimation task, and shared useful information between two sub-tasks to guide deeper feature extraction via a cross-connection, and finally fused the deeper features to estimate 3D hand pose. Chen et al. [26] firstly exploited an initially estimated hand pose as guided information to extract effective joint features. Secondly, joint features belonging to the same finger were integrated to form finger features, and then finger features were further integrated to regress 3D hand pose. The entire estimation process was performed iteratively. Dai et al. [25] further extended the hierarchical idea of 3D hand pose estimation from color images, which improved the performance of 3D hand pose estimation from color images.

To further improve the performance of 3D hand pose estimation based on RGB images, a novel hierarchical estimation method based on hand structure is proposed after analyzing the hand structure, which is most similar to the method proposed in [26]. Here, it is necessary for us to emphasize the difference between them. First, Ref. [26] is based on depth images, while our work is based on RGB images, which is essentially different from Chen et al. [26]. Second, it first extracts joint features and then integrates them into finger features, while our work directly extracts finger features through a hierarchical method, which reduces the depth of the network while ensuring accuracy. Again, ours works without estimated 3D hand pose to guide the feature extraction, which eliminates the need for iteration and reduces the complexity of the network. Finally, unlike the model proposed by Chen et al. [26], where the Palm is connected to five fingers, our method connects only the Palm and the middle finger. Subsequent experimental analysis shows that the Palm connected with multiple fingers simultaneously affects the estimation accuracy.

## 3. Methodology

### 3.1. Overview

**Figure 1:**
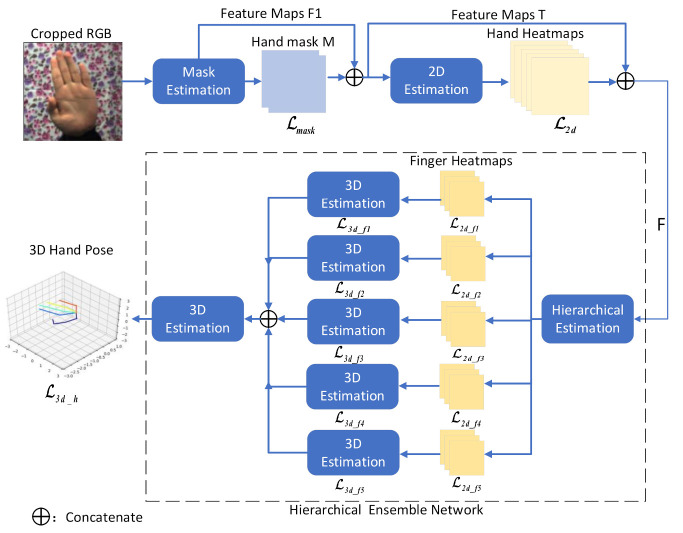
Schematic diagram of proposed 5LENet framework. Our 5LENet receives a cropped RGB image as the input to estimate hand mask. Then, the 2D hand heatmaps are estimated according to the features of RGB image and hand mask. Finally, 3D hand pose is estimated through the hierarchical ensemble network in the dotted box.

Our target is to infer a 3D hand pose from a single RGB image. To achieve this goal, a novel hierarchical network called Five-Layer Ensemble CNN (5LENet) is proposed, as illustrated in Figure 1. First, a localization segmentation network is used to locate the salient hand and crop hand image as the input of our network, which will be briefly described in Section 3.2. Next, 3D hand pose is estimated, and the whole estimating process can be divided into three stages: hand mask estimation stage, 2D hand heatmap estimation stage, and 3D hand pose estimation stage. The first stage obtains a hand mask by processing the cropped image. The second stage connects the hand mask and the intermediate image features to estimate the 2D hand heatmaps. The last stage connects hand heatmaps with the intermediate image features to estimate single finger heatmaps hierarchically and then estimates their corresponding 3D finger pose, finally fusing the extracted 3D finger feature information to estimate 3D hand pose. We will introduce the first and second stages in Section 3.3. The method of estimating a single finger pose based on the hierarchical network and fusing 3D feature information of five fingers to estimate 3D hand pose will be focused on in Section 3.4.

### 3.2. Localization and Segmentation Network

The goal of the localization and segmentation network is to locate the salient hand position of the RGB image and obtain the hand bounding box to crop the hand image. A lightweight Convolutional Pose Machine (CPM) [29] is used as the localization and segmentation network, which exploits a series of convolutional operations to encode the spatial structure of the RGB image, namely, a 2-channel hand mask, and calculates the center of mass of the hand mask. Then, the hand area is cropped around the center of mass and resized to 256×256.

### 3.3. 2D Pose Estimation Network

Two-dimensional pose estimation is the key to 3D pose estimation. For the sake of accurately estimating 2D hand pose, we first use a lightweight VGG-19 [30] mask estimation network to encode the input cropped image. Both 128-channel image features F1 and 2-channel hand mask M are extracted by convolution. The Boolean hand mask distinguishes the hand and background areas, which is beneficial to the subsequent 2D hand pose estimation.

Based on the discussion in [31], the promotion of regressing 2D keypoint heatmaps is better than direct regressing 2D keypoint coordinates. Therefore, we employ the method of regressing 2D keypoint heatmaps to estimate 2D pose, taking 130-channel features T as the input of the 2D pose estimation network, which consists of a 2-channel hand mask M and 128-channel image features F1, to output 2D heatmaps of 21 keypoints. The 2D pose estimation network consists of five sub-networks, in which 21-channel hand heatmaps output by the previous sub-network and 130-channel image features T are connected to form 151-channel features as the input of the latter sub-network, then the location of 21 keypoints are refined iteratively. We take the 21-channel 2D heatmaps estimated by the final sub-network as the final output.

### 3.4. Hierarchical Ensemble Network

The hierarchical ensemble network is the main part of our proposed 5LENet. It first adopts a hierarchical estimation network to divide hand features into five layers: the thumb layer, index finger layer, middle finger layer, ring finger layer, and pinky finger layer, respectively, and each finger heatmap is estimated separately, of which only the middle finger layer adds the Palm joint. Based on the 2D finger heatmap features, deeper-level 3D finger features are extracted to estimate 3D finger pose, and finally, five layers of 3D finger features are assembled to estimate 3D hand pose. This hierarchical ensemble method is conducive to extracting deeper-level and more effective hand feature information, thereby improving the performance of 3D hand pose estimation.

#### 3.4.1. Hierarchical Estimation Network

**Figure 2:**
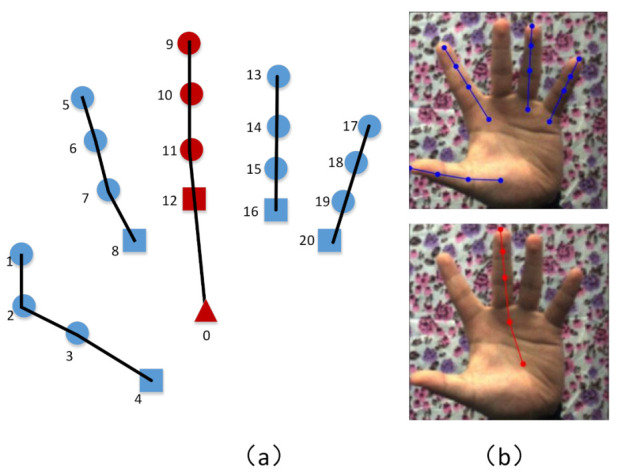
The diagram and examples of hand keypoint division. (**a**) Skeleton graph of 21 hand keypoints, in which triangle, square, and circle represent Palm, the metacarpophalangeal joint, and the phalangeal joint, respectively. The middle finger containing the Palm is marked in red, and others are marked in blue. (**b**) An example diagram of different finger keypoints from the real dataset Stereo Hand Pose Tracking Benchmark (STB).

The target of our hierarchical estimation network is to estimate 2D heatmaps of five fingers separately, and the division of hand keypoints is shown in Figure 2a. Among the five fingers, only the middle finger is connected to the Palm, which is distinguished by different colors (blue and red) in Figure 2a. Figure 2b shows the examples of the keypoint partition of each finger from the samples of the real dataset Stereo Hand Pose Tracking Benchmark (STB).

**Figure 3:**
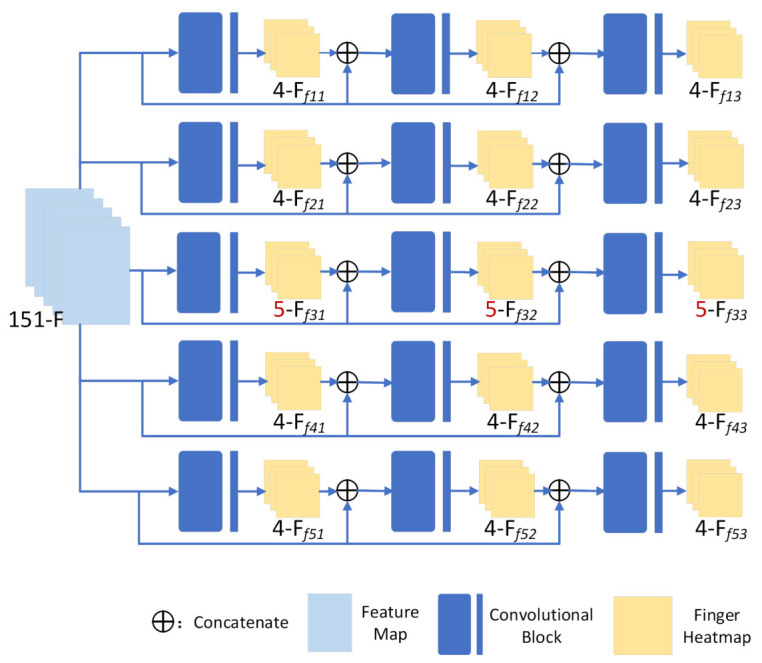
The architecture of the hierarchical estimation network. Five 2D single finger heatmaps are estimated by the network, of which n-$F_{fij}$ represents the n-channel 2D heatmaps of the ith finger estimated by jth layer.

The hierarchical estimation network decomposes hand features into five layers according to five different fingers to estimate the 2D heatmaps of each corresponding finger. As illustrated in Figure 3, there are five layers in the whole network, and each layer is composed of three sub-networks. Taking the first layer (thumb layer) as an example, the 4-channel thumb heatmaps output by the previous sub-network are connected with 151-channel features F to form 155-channel features as the input of the next sub-network, which optimizes the location of thumb keypoints iteratively. Then, we take 4-channel 2D thumb heatmaps $F_{f13}$ estimated by the final sub-network as the output for the thumb layer. The 2D heatmap estimation process of other fingers is the same as that of the thumb layer, the only difference being that the number of output channels of middle finger is 5, while others are 4.

#### 3.4.2. 3D Pose Estimation Network

Based on the five 2D finger heatmaps estimated by the hierarchical network, we further estimate the 3D pose of each finger, and then 3D hand pose is regressed from the concatenated 3D finger features of the last FC layer of all 3D finger pose estimation. For regression, we first feed the integrated 3D features into two 512-dimensional FC layers with a dropout rate of 0.2 and then regress the 3D coordinates of 21 keypoints.

This paper follows the representation in [12], using relative normalized coordinates. $W^{rel}={\left\{w_{j}^{rel}\right\}}_{j=1}^{J}\in \Lambda_{3D}$ represents 3D keypoint coordinates, where ${\;\Lambda }_{3D}$ and $w_{j}^{rel}$ indicate the J×3-dimensional hand joints space and the relative normalized coordinates of the jth keypoint, respectively. The 3D canonical coordinates $W^{c}$ and 3D rotation matrix R are estimated parallelly within the canonical frame, and then $W^{rel}$ is obtained indirectly, of which R is equivalent to predicting the viewpoint of a given sample concerning the canonical frame.

(1) $$W^{rel}=W^{c}\cdot R^{T}$$

### 3.5. Loss Functions

#### 3.5.1. Estimation Loss of 2D Pose

Before estimating 2D hand pose, the cropped image is fed to the mask estimation network to estimate a 2-channel hand mask M. The standard softmax cross-entropy loss is adopted to calculate the hand mask loss $L_{mask}$, and it is defined as follows:

(2) $$L_{mask}=\;-\sum \log\left(\frac{e^{s_{y}}}{\sum_{k}e^{s_{k}}}\right)$$

where y is the corresponding label of hand mask, and $s_{k}$ is the output score of the kth label, where the mask is a binary map, $k\in \left\{0,1\right\}$.

During the process of full 2D hand pose estimation, the $L_{2}\;\mathrm{loss}$ is imposed on the 2D heatmaps of 21 keypoints to calculate the estimation loss of 2D hand pose $L_{2d}$, and the loss function is defined as follows:

(3) $$\;L_{2d}=\overset{J=21}{\underset{j=1}{\sum }}{||h_{j}^{pre}-h_{j}^{gt}||}_{2}$$

where $h_{j}^{pre}$ and $h_{j}^{gt}$ represent the estimated and ground truth heatmaps of the jth keypoint, respectively. The ground truth heatmaps for the jth keypoint are generated by applying 2D Gaussian centered at its ground truth 2D keypoint location.

#### 3.5.2. Loss of Hierarchical Estimation

The loss of hierarchical estimation $L_{L}$ is the sum of the loss of five 2D finger heatmaps; $fi\;\left(i=1,2,3,4,5\right)$ represents the corresponding finger, where f1 represents the first finger (thumb), and the others are the index finger, middle finger, ring finger, and pinky. The loss of hierarchical estimation $L_{L}$ is summarized as follows:

(4) $$L_{L}=\overset{5}{\underset{i=1}{\sum }}{||h_{fi}^{pre}-h_{fi}^{gt}||}_{2}$$

where $h_{fi}^{pre}$ and $h_{fi}^{gt}$ denote the estimated and ground truth heatmaps of the  ith finger.

#### 3.5.3. Estimation Loss of 3D Pose

The estimation loss of 3D pose $L_{3d}$ is composed of six parts, which are the 3D pose estimation loss of five fingers $L_{3d\_fi\;}\;\left(i=1,2,3,4,5\right)$ and the full 3D hand pose loss $L_{3d\_h}$, where fi represents the thumb, index finger, middle finger, ring finger, and pinky, respectively, of which only the middle finger contains the Palm joint. $L_{3d\_fi\;}$ is used as supervision to guide the network to extract specific 3D features of each finger and promote a more accurate 3D estimation of the whole hand, and $L_{3d\_h}$ is used as supervision to guide the predictions of the whole hand. We employ $\;L_{2\;}\mathrm{loss}$ to calculate the loss of the corresponding part of 3D canonical coordinates $W^{c}$ and 3D rotation matrix R. They are defined as follows:

(5) $$L_{3d\_fi}={||W_{fi}^{pre\_c}-W_{fi}^{gt\_c}||}_{2}+R_{fi}^{pre}-{||R_{fi}^{gt}||}_{2},\;i=\left(1,2,3,4,5\right)$$

(6) $$L_{3d\_fi}={||W_{fi}^{pre\_c}-W_{fi}^{gt\_c}||}_{2}+{||R_{fi}^{pre}-R_{fi}^{gt}||}_{2},\;i=\left(1,2,3,4,5\right)$$

(7) $$L_{3d}=\overset{5}{\underset{i=1}{\sum }}L_{3d\_fi}+L_{3d\_h}$$

where $W_{fi}^{pre\_c}$ and $W_{h}^{pre\_c}$ denote the estimated 3D canonical coordinates of the  ith finger and the full hand, and $W_{fi}^{gt\_c}$ and $W_{h}^{gt\_c}$ are their corresponding 3D labels. $R_{fi}^{pre}$ and $R_{h}^{pre}$ denote the estimated 3D rotation matrix of the ith finger and the full hand, and $R_{fi}^{gt}$ and $R_{fi}^{gt}$ are their ground truth.

#### 3.5.4. Total Loss of Network

Because the loss value of hand mask is too large, we add a weight ratio  λ  to this item to reduce its loss value. Experimental results show that the model achieves the best performance when λ=0.05, and the total loss of our entire network $L_{total}$ is defined as follows:

(8) $$L_{total}=\;\lambda *L_{mask}+L_{2d}+L_{L}+L_{3d}$$

## 4. Experiments

### 4.1. Datasets and Metrics

#### 4.1.1. OneHand10K

OneHand10K [32] is a dataset based on single-handed RGB images with different resolutions, which is composed of 10,000 images for training and 1703 images for testing. All samples are annotated with 2D keypoint locations and hand masks. The early work of Liu et al. [27] proved the superiority of using OneHand10K to train the localization segmentation network, so we follow their method to exploit ground truth hand masks provided by OneHand10K to train our localization segmentation network and promote the model’s adaptability to the real environment.

Because the image resolution of this dataset is not uniform, it is necessary to adjust and fill the original image $I\in {\mathbb{R}}^{w\times h\times 3}\;$, where w and  h  denote the width and height of the image, respectively. We first adjust the resolution of all color images and corresponding masks with a ratio 𝓉, then fill the m×m image with the resized color image from the left-top corner, where m=320, and fill the remaining empty region with the gray value (128,128,128). Similar procedures are performed for the hand mask, but filled with zeros.

(9) $$𝓉=\min\;\left(\frac{m}{w},\frac{m}{h}\right)$$

#### 4.1.2. RHD

Rendered Hand Pose Dataset (RHD) [12] is a fully synthetic dataset with rendered hand images, which is composed of 41,258 training images and 2728 testing images with the resolution of 320×320. All samples come with full annotation of 2D and 3D labels of 21 keypoints and additionally corresponding mask labels and depth images. The dataset is highly challenging due to the diverse visual scenery, illumination, and noise. We use all labels except the depth images to train the entire network.

#### 4.1.3. STB

Stereo Hand Pose Tracking Benchmark (STB) [33] is a real dataset, which contains two different subsets of image resolution of 640×480: STB-BB and STB-SK. The images in STB-BB and STB-SK are captured by the Point Grey Bumblebee2 stereo camera and the Intel F200 depth camera, respectively. Both subsets provide 2D and 3D labels of 21 keypoints. In our experiments, we only use the STB-BB subset for 5LENet training and testing. STB-BB has 12 sequences totaling 36,000 images, which includes six different backgrounds. Following the training and evaluation scheme in [12], 10 of these sequences are used as the training set, and the remaining are the testing set. Due to the domain gap between the synthetic and real images, the model trained by RHD cannot adapt well to the real environment. Thus, we use 2D and 3D labels of the STB-BB subset to fine-tune the model pre-trained by RHD to promote greater adaptability of the model.

#### 4.1.4. Evaluation Metrics

As proposed in [12], we quantitatively evaluate the 3D hand pose estimation performance with two metrics. The first metric is end-point-error (EPE), the Euclidean distance between the predicted 3D keypoint coordinates and the ground truth, and the second metric is the area under the curve (AUC) on the percentage of correct keypoints (PCK) under different error thresholds σ in the 3D space. They are defined as follows:

(10) $$PCK_{\sigma }=\;\frac{1}{J}\overset{J}{\underset{j=1}{\sum }}\delta \left(||w_{j}^{pre}\;-\;w_{j}^{gt}||\;<\sigma \right)$$

(11) $$AUC_{J}\;=\;\int PCK_{\sigma }^{J}\;$$

where $w_{j}^{pre}$ represents the estimated 3D coordinates of the jth keypoint, $w_{j}^{gt}$ is its corresponding 3D labels, and δ is an indicate function.

### 4.2. Experimental Details

Our method is implemented with TensorFlow [34], and an Adam optimizer [35] is used to train the entire network. All experiments are conducted on a single GPU server with CUDA 8.0 NVIDIA RTX2080Ti.

The input of our network is a cropped RGB image preprocessed by localization and segmentation network, which is trained by the OneHand10K in Section 4.1.1. Its training batch is 8, and the initial learning rate is set to $5\times 10^{-5}$; the learning rate decreases at a rate of 0.1 every 10 K iterations, with a total of 40 K iterations.

The entire 5LENet is trained in an end-to-end manner. We first use RHD to pre-train the network; the training batch is 8, and the initial learning rate is $5\times 10^{-5}$. The learning rate decreases by 0.3 for every 50 K iterations, with a total of 300 K iterations. Then, the STB is used to fine-tune the pre-training model to improve its adaptability to the real environment. Except for the different learning rate settings, the remaining training parameters are the same as the pre-training process. The learning rate is $1.5\times 10^{-5}\;$ for the first 50 K iterations and then decays every 50 K iterations with the decay ratio 0.3; after 250 K iterations, the network is trained at $3.645\times 10^{-8}$ until the end.

### 4.3. Ablation Study

In order to verify the effectiveness of different components of our 5LENet, we conduct extensive ablation studies on the number of network layers, the newly added 3D finger pose constraints, and Palm-to-finger connection problems. For clarity of discussion, we mark the thumb, index finger, middle finger, ring finger, and pinky as T, I, M, R, and P, respectively, and the center of the palm as Palm. Note that the setting of all ablation experimental parameters in this paper is consistent, and the model is pre-trained by RHD and then fine-tuned by STB using the same OneHand10K-trained localization segmentation network to preprocess the input image.

#### 4.3.1. Effectiveness of Five-Layer Network

**Figure 4:**
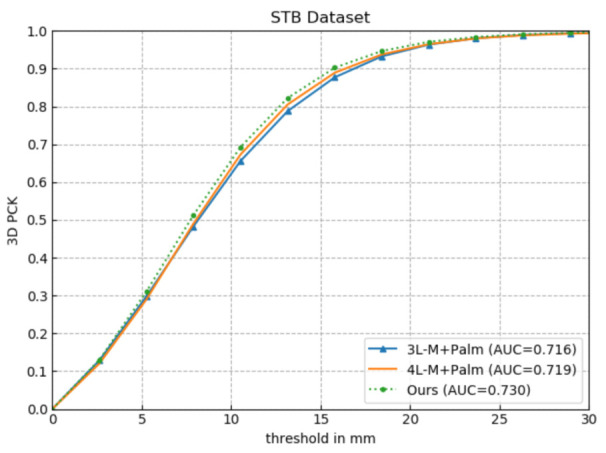
Comparative experiment of the effectiveness of the five-layer network.

To verify the superiority of our five-layer network, in this experiment, we uniformly use a hand model with the Palm connected to the middle finger and add 3D finger constraints. Only the hand hierarchical structure is changed, which is divided into five layers, four layers, and three layers, represented by Ours, 4L-M+Palm, and 3L-M+Palm, respectively. The hand division structure of the four-layer and three-layer layers is T, I, M, R/P, and T/I, M, R/P. The experimental results of using the evaluation metrics in Section 4.1.4 to evaluate Ours, 4L-M+Palm, and 3L-M+Palm on the STB dataset are shown in Figure 4 and Table 1.

As illustrated in Figure 4, Ours achieves an AUC of 0.730 within the error threshold 0–30 mm, which improves by 1.96% and 1.53%, respectively, compared with 0.716 of 3L-M+Plam and 0.719 of 4L-M+Palm. From Table 1, we can observe that as the number of network layers increases, the estimation error gradually decreases, which successfully verifies the effectiveness of the five-layer network to extract deeper and more representative features, thereby further improving the accuracy of 3D hand pose estimation.

#### 4.3.2. Effectiveness of Newly Added 3D Finger Pose Constraints

**Figure 5:**
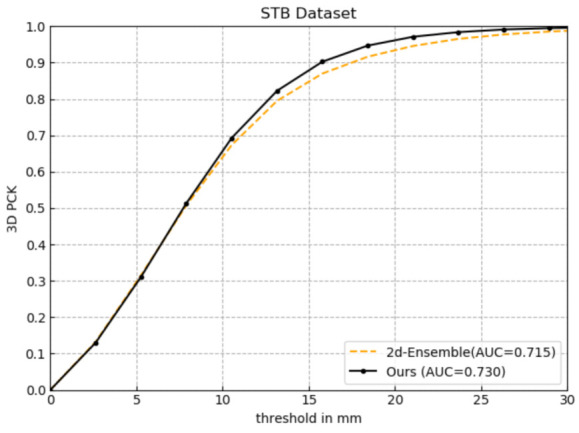
Comparative experiment of the effectiveness of newly added five 3D finger pose constraints.

In this section, the effectiveness of another component of our proposed method will be demonstrated: the newly added 3D finger pose constraints. The basic network architecture is kept the same, among which Ours adds five 3D finger pose constraints, while 2d-Ensemble directly fuses the 2D finger feature information to estimate full 3D hand pose. Ours and 2d-Ensemble are evaluated on STB with the metrics in Section 4.1.4; the experimental results are shown in Figure 5 and Table 2.

As shown in Figure 5, compared with 2d-Ensemble 0.715, the AUC of ours has improved 0.015 within the error threshold 0-–30mm. Table 2 displays the 3D hand pose estimation errors of 2d-Ensemble and Ours. Compared with 2d-Ensemble, the average endpoint error and median endpoint error of Ours are reduced by 0.511 (mm) and 0.110 (mm), respectively, which demonstrates that the newly added constraints can effectively improve the accuracy of 3D hand pose estimation.

#### 4.3.3. Effectiveness of Connecting Palm with a Single Finger

**Figure 6:**
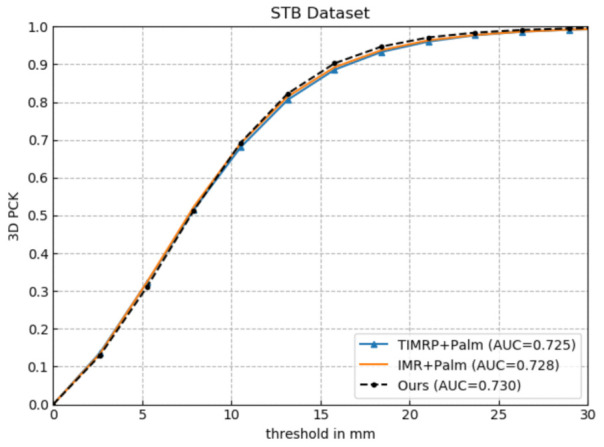
Comparative experiment of the effectiveness of the Palm connecting with a finger.

In order to validate the effectiveness of the Palm connected with a single finger for 3D hand pose estimation, we keep the five-layer network and newly added five 3D finger pose constraints the same and change the number of fingers connected to the Palm, of which Ours represents the Palm connected to the middle finger, and TIMRP+Palm and IMR+Palm represent the Palm connected with all fingers, and connected with the index finger, middle finger, and ring finger, respectively. The metrics mentioned in Section 4.1.4 are used to evaluate them on STB. Figure 6 and Table 3 show their experimental results.

As shown in Figure 6, Ours achieves the best performance within the given error threshold, and its AUC value reaches 0.730. From Table 3, we can find that as the number of fingers connected to the Palm increases, the estimation accuracy of the network decreases instead. The estimated result shows that the structure with the Palm connected with multiple fingers cannot promote estimation accuracy; on the contrary, it will affect the accuracy. Thus, this experiment demonstrates that the Palm connected with a single finger helps to boost the performance of estimation.

#### 4.3.4. Effectiveness of Connecting Palm with Middle Finger

**Figure 7:**
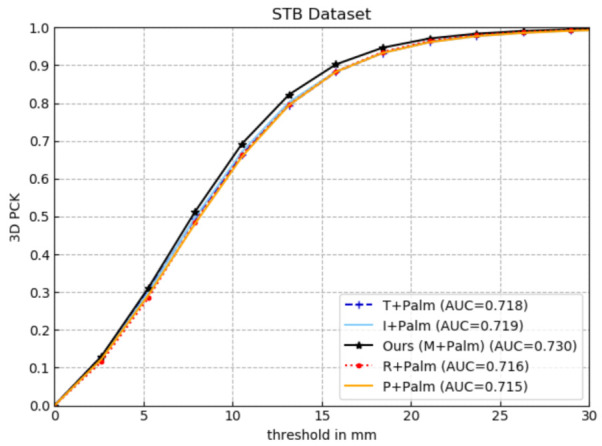
Comparative experiment of the effectiveness of the Palm connecting with middle finger.

Since the validity of the Palm connected with a single finger has been verified in Section 4.3.3, this section will further demonstrate the effectiveness of the Palm connected with the middle finger. The five-layer network and the newly added five 3D finger pose constraint are unchanged, and the Palm is changed to connect with the thumb, index finger, middle finger, and pinky, respectively, which are represented by T+Palm, I+Palm, Ours (M+Palm), R+Palm, and P+Palm. Similarly, using the same metrics in Section 4.1.4 to evaluate them on the STB dataset, the experimental results are shown in Figure 7 and Table 4.

It can be seen from Figure 7 that the AUC value of Ours (M+Palm) within the error threshold is higher than that of other networks, while the estimation performance of others is considerably close. In Table 4, Ours (M+Palm) achieves the minimum error both in the average endpoint error and the median endpoint error. The reason why Ours (M+Palm) achieves the highest estimation accuracy is that the Palm and the middle finger are more closely related with each other than other fingers, according to the topological features of the hand.

### 4.4. Comparison with the State-of-the-Art Methods

**Figure 8:**
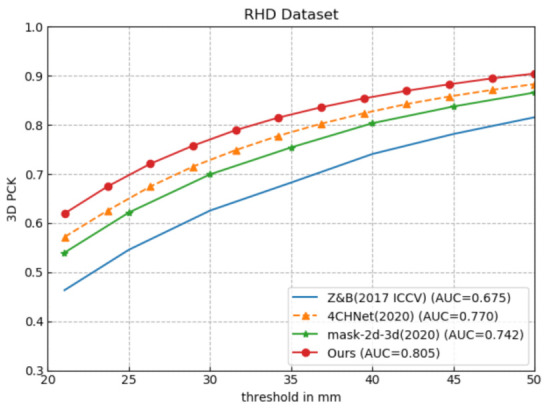
Comparison with the state-of-the-art methods on Rendered Hand Pose (RHD) dataset [12].

**Figure 9:**
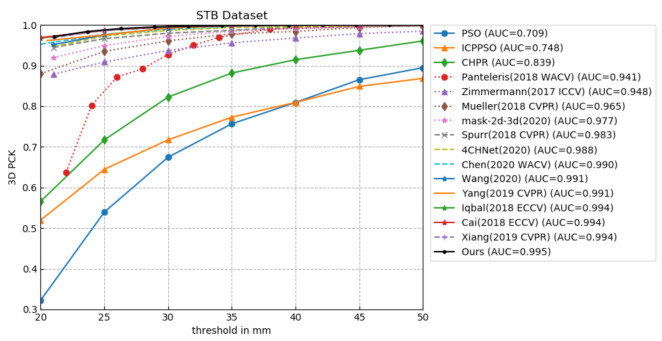
Comparison with the state-of-the-art methods on STB dataset [33].

As shown in Figure 8 and Figure 9, under the same evaluation conditions and criteria, we perform a quantitative comparison of our proposed 5LENet with other previous competitive methods on RHD and STB datasets. Since the 3D hand pose estimation method based on depth image is inconsistent with the evaluation metric based on color image method, here, we do not make a quantitative comparison with the most similar work [26] to ours.

As illustrated in Figure 8, our 5LENet outperforms state-of-the-art methods [12,25,27] by a large margin on the RHD dataset. The AUC value of Ours reaches 0.805 within the error threshold 20–50 mm, which is improved by 19.26%, 8.49%, and 4.55%, respectively, compared to Zimmermann et al. [12], mask-2d-3d [27], and 4CHNet [25]. All experimental results of the competitive methods are derived from the corresponding literature.

Figure 9 shows the comparisons with state-of-the-art methods [8,12,13,14,15,19,25,36,37,38,39,40,41,42] on the STB dataset; Ours represents the proposed 5LENet. It is worth noting that in addition to the pose estimation error, the process of hand positioning will also produce the error, so the methods involved in the comparison also need to add the corresponding localization error if they do not have it. As presented in Figure 9, our method achieves the highest AUC value 0.995 within the error threshold of 20-50mm; compared with 0.948 of Zimmermann et al. [12] and 0.988 of 4CHNet [25], the performance improves by 0.047 and 0.007, respectively, where 4CHNet [25] employs a hierarchical network to divide hand features into two layers of palm and finger features. The high precision estimation result of ours again verifies the effectiveness and advancement of the five-layer network and five newly added 3D finger constraints. There is a substantial improvement compared with the 0.991 of Wang et al. [13] and Yang et al. [39], while Wang et al. [13] use an RHD-trained localization segmentation network to crop the hand area, which cannot adapt well to the real environment compared with the OneHand10K-trained network. Yang et al. [39] achieve image synthesis and pose estimation by learning disentangled representations of hand poses and hand images, but to a certain extent, the disentangling process will lead to the missing information that helps to generate useful data. Additionally, although the estimation results of Iqbal [42], Xiang [41], and Cai et al. [15] are significantly close to ours, it is necessary to emphasize the difference between theirs and ours here. Among them, in [41,42], the hand location error is not considered, and in [15], the paired depth and RGB image are required, which are rather difficult to collect, so our network is superior to theirs to a certain extent. The results from competing methods are derived from the corresponding literature.

### 4.5. Qualitative Results

**Figure 10:**
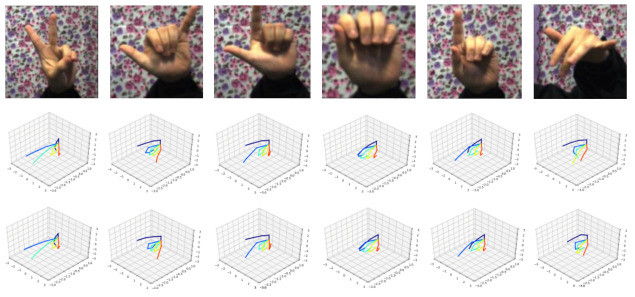
Qualitative results for STB dataset [33].

**Figure 11:**
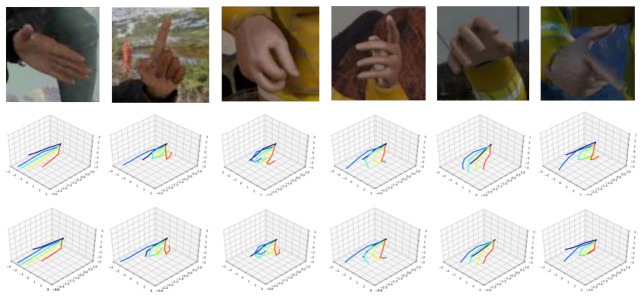
Qualitative results for RHD dataset [12].

Some qualitative results of 5LENet on STB and RHD can be seen in Figure 10 and Figure 11. For each dataset, the first row represents the cropped input RGB image, the second row shows the estimated 3D hand pose results of the proposed 5LENet, and the third row is the ground truth. It is observed that the estimation of 3D hand poses is visually very similar to the ground truth ones, and for some self-occluded hand poses, as shown in Figure 10, our 5LENet also has comparable estimated performance. Due to the complexity and challenges of the synthetic dataset RHD, as shown in Figure 11, though the 3D hand poses predicted by 5LENet are similar to the ground truth, there are still some gaps. 

## 5. Conclusions

In this paper, a novel five-layer ensemble CNN (5LENet) is proposed for accurate 3D hand pose estimation from a single RGB image. Based on hierarchical thinking, we designed a hierarchical network to decompose the full hand pose estimation into five single-finger pose tasks. It can not only promote a more accurate 2D finger pose estimation but also can further optimize the estimation of 3D finger hand pose. Then, the 3D finger features optimized in the estimation process are fused to estimate the full 3D hand pose. At the same time, 5LENet leverages five newly added 3D finger pose constraints to extract more representative features to improve estimation accuracy. Furthermore, we also build a hand model with the Palm connected to the middle finger, which can further help to improve the estimation performance. We make quantitative comparisons with other state-of-the-art methods on RHD and STB datasets. The high-precision 3D estimation results demonstrate the advancement and superiority of our proposed network.

## Figures and Tables

**Table 1:** Error analysis of five-layer network effectiveness.

Network	EPE Median (mm)	EPE Mean (mm)	AUC
3L-M+Palm	8.408	8.957	0.716
4L-M+Palm	8.253	8.842	0.719
Ours	7.937	8.492	0.730

**Table 2:** Error analysis of newly added five 3D finger pose constraints effectiveness.

Network	EPE Median (mm)	EPE Mean (mm)	AUC
2d-Ensemble	8.047	9.003	0.715
Ours	7.937	8.492	0.730

**Table 3:** Error analysis of connecting Palm with a single finger.

Network	EPE Median (mm)	EPE Mean (mm)	AUC
TIMRP+Plam	8.032	8.672	0.725
IMR+Palm	7.949	8.575	0.728
Ours	7.937	8.492	0.730

**Table 4:** Error analysis of connecting Palm with middle finger.

Network	EPE Median (mm)	EPE Mean (mm)	AUC
t+palm	8.258	8.858	0.718
I+Palm	8.195	8.852	0.719
R+Palm	8.351	8.940	0.716
P+Palm	8.375	8.989	0.715
Ours (M+Palm)	7.937	8.492	0.730

## Data Availability

Data is contained within the article.
